# The role of primary care providers in patient activation and engagement in self-management: a cross-sectional analysis

**DOI:** 10.1186/s12913-016-1328-3

**Published:** 2016-03-11

**Authors:** Carmen Alvarez, Jessica Greene, Judith Hibbard, Valerie Overton

**Affiliations:** Department Community-Public Health, Johns Hopkins University School of Nursing, 525 N. Wolfe St, Baltimore, MD 21201 USA; School of Nursing, George Washington University, Washington, DC USA; Health Policy Research Group, 1209 University of Oregon, Eugene, Oregon 97403, Eugene, OR USA; Fairview Medical Group, Minneapolis, Minnesota USA

**Keywords:** Primary care providers, Self-management support, Patient activation

## Abstract

**Background:**

The increasing burden of chronic illness highlights the importance of self-care and shifts from hierarchical and patriarchal models to partnerships. Primary care providers (PCPs) play an important role in supporting patients in self-management, enabling activation and supporting chronic care. We explored the extent to which PCPs’ beliefs about the importance of the patients’ role relate to the frequency in which they report engaging in collaborative and partnership-building behaviors with patients.

**Methods:**

PCPs’ beliefs were measured using the Clinician Support for Patient Activation Measure (CS-PAM). We also assessed whether PCPs’ CS-PAM scores were positively associated with changes in their patients’ Patient Activation Measure (PAM) scores. Participants included 181 PCPs from a single accountable care organization in Minnesota who completed an online survey. We conducted bivariate analyses and multivariate regression models to examine relationships between CS-PAM and PCP self-management support behaviors and changes in level of patient activation.

**Results:**

PCPs with high CS-PAM scores were much more likely to engage in supportive self-management and patient behavior change approaches, such as involving the patient in agenda-setting, problem-solving, and collaboratively setting behavioral goals, than were PCPs with low CS-PAM scores. More positive PCPs’ belief in the patients’ role in self-management was positively correlated with improvements in their patients’ level of patient activation.

**Conclusions:**

More positive PCP beliefs about the patients’ role in self-management was strongly related to PCP behaviors geared towards increasing patient activation.

## Background

Internationally, there is increased emphasis on chronic care management, which represents a complex interplay between patient, provider, and health care system factors [1]. In the United States, health care reforms driven by the Affordable Care Act (ACA) call for changes that move away from the fee-for-service reimbursement model to pay-for-perfomance. The fee-for-service reimbursement model rewards quantity of medical services; instead pay-for-performance ties payment more closely with improved patient outcomes, improved quality, and restrained costs [2–4]. These changes signal transformational changes for practitioners and delivery systems as well as for patients’ values, attitudes and beliefs. Delivery systems are recognizing that patients are an important resource in the healthcare process as they carry out the day-to-day care management tasks, decide whether or not to follow through on treatment regimens, and make necessary life-style adjustments to improve their health [5]. Without the patients’ engagement, even with best practices on the part of healthcare providers, it is very difficult to achieve optimal health outcomes and constrain costs.

Primary care providers (PCPs) are on the front lines of this change. Their role in working with patients is pivotal in achieving these ends; however, for many PCPs supporting patient self-management and greater patient activation has not been part of their training and, moreover, is not part of how they understand their role as clinicians. Some do not embrace the strategies that involve partnering with patients to improve self-management and supporting patient behavior change, and do not see this as an important part of their job [6, 7].

This paper explores provider beliefs about the importance of the patient role and how those beliefs are related to their reported behaviors in clinical encounters. There is a scant literature linking patients’ positive perceptions of provider support for self-management and patients’ own engagement in self-management behaviors [8–10]. Even fewer studies have examined provider beliefs about the importance of supporting patients in managing their health conditions [11]. We seek to build on and contribute to the literature by measuring provider beliefs about the patient role and by examining the degree to which those beliefs are positively associated with provider behaviors in the medical encounters. If clinician beliefs underlie behaviors that result in greater or lesser patient activation, then this may be a point of leverage for making changes that can ultimately improve outcomes of care.

In this study we explore how PCPs in a single Accountable Care Organization–Fairview Health Services in Minnesota–view the importance of the patients’ role, using a relatively new measure of clinician support for patient activation [6]. We examine how PCPs’ views on the patients’ role relate to the frequency that PCPs engage in collaborative and partnership-building behaviors with patients to support self-management and behavioral change. Further, we examine whether the providers’ views on the patients’ role are positively associated with changes in the patient activation scores (PAM scores) among their patient panels over time. Patient activation refers to having the knowledge, skill, and confidence to manage one’s own health and health care and is assessed using the 13-item Patient Activation Measure (PAM) [12].

## Methods

This study examines the relationship between clinician support of patient activation (CS-PAM) and three sets of outcomes: clinician behaviors that support patient self-management, clinician behaviors in support of behavior change, and actual changes in patient activation measure scores (PAM) among a PCP’s patient panel. Thus we present two types of analyses: Our primary analysis—exploring the relationship between CS-PAM and clinician behaviors—is cross-sectional; our supplementary analysis examines the relationship between CS-PAM and changes in patient activation.

The institutional review boards of the University of Oregon, University of Minnesota and George Washington University approved the study procedures. The study was conducted in collaboration with the Fairview Health System in the summer of 2013. This health system includes 44 primary care clinics; PCPs from these clinics completed an online survey. The online survey was based on qualitative findings from a preliminary study exploring provider perceptions about a new compensation model implemented within the Fairview system [12]. Survey items included items about quality of care, productivity, and strategies providers may use to influence patient health behavior [13].

For some analyses, we used data extracted from Fairview’s electronic health record to compute descriptives of PCPs’ patient panels. Additionally, to compute the average change in activation for a PCP’s panel of patients, we used a panel of 10,957 patients who had two PAM scores in consecutive years between 2010 and 2012. For this portion of the analysis, we only included those PCPs who had at least 20 patients on their panels with two PAM scores.

### Survey items

#### Independent variable

##### Clinician Support for patient activation

We measured providers’ beliefs about the patients’ role in their own care using the CS-PAM (Appendix). This 13-item scale was modified from the Patient Activation Measure, which asks patients how much they agree or disagree with statements related to how they manage their health. In the CS-PAM, clinicians respond to how important to them as clinicians are each patient skill, areas of knowledge, or beliefs. For example, clinicians were asked, “How important is it to you that your patients with chronic conditions can follow through on medical treatments they need to do at home?” PCPs answer using a four-point scale in which 1 = not important and 4 = extremely important. Scores are translated into a 1–100 scale, based upon Rasch methods [14], with higher scores indicating more positive beliefs about the importance of patient knowledge and involvement in his/her care. The original version of this measure (14-items) was used among a sample of clinicians from the US, UK, and The Netherlands [6, 15]. The CS-PAM demonstrated high reliability in this sample (Cronbach’s alpha α = .97).

#### Dependent variables

##### Clinicians’engagement in chronic illness management support behaviors

The first set of 7 items—adapted from the Patient Assessment for Chronic Illness Care (PACIC)—were designed to assess the degree to which clinicians engage in partnership-building behaviors with patients around self-management [8]. An example of an original item from the PACIC is – “…when I received care from my chronic conditions, I was: asked for my ideas when we made a treatment plan.” The items where then adapted to be relevant for providers; for example, one item asks: “Over that last month when you treated patients with chronic conditions, how often did you make sure patients were involved in setting the agenda for the visit? (1 = almost never, 4 = almost always).

##### Using specific strategies to support patient behavior change

The second set of 8 items was derived from qualitative interviews with Fairview clinicians and is based on their descriptions of their strategies for supporting their patients’ behavior changes. For example, “When a patient is not making needed behavioral changes to meet quality metrics, how often do you … have frank and sometimes difficult conversations with a patient about his or her behaviors?” (1 = never, 5 = very often). These 8-items and the 7-items adapted from the PACIC were all treated as individual items.

The final dependent variable was the average amount of change in patient activation score a PCPs’ patient panel had over 1 year. The “patient panel” is the collective term that refers to the patients seen by a provider over 1 year. Given that Fairview routinely collects patient activation data on patients, this latter outcome measure was derived from data from the electronic medical records; thus, the patient activation scores did not come from the same data source as the clinician survey.

### Statistical analyses

We examined the relationships between provider characteristics and CS-PAM score, and providers’ patient panel characteristics (percent women, average age, average risk score, average mean income for ZIP Code), and CS-PAM score. None of the patient panel characteristics were related to the CS-PAM, and for the sake of parsimony, we did not include the patient panel characteristics in further analysis. To examine the relationship between CS-PAM scores and the dependent variables (Clinician engagement in chronic illness management support behaviors and using specific strategies to support patient behavior change), we initially conducted bivariate analyses. Specifically, we examined what percentage of PCPs in each CS-PAM tercile “almost always” or “very often” (depending on the set of items) engaged in the specific behavior. For the third dependent variable, we examined the relationship between the CS-PAM scores and the providers’ mean patient panel change in PAM scores.

We then developed multivariate regression analyses to examine the relationships between CS-PAM and the dependent variables, controlling for the following provider characteristics: gender, provider age, years of work at Fairview, and type of provider. For the first two sets of dependent variables, which were treated as dichotomous, we developed logistic regression models, and for the last dependent variable, we used ordinary least squares regression.

## Results

Of the 266 PCPs invited to participate in the survey, 181 completed the survey, for a response rate of 68 %. For analyses using the patient panel data, the sample size of clinicians was substantially smaller (*n* = 64).

PCPs were predominately female (60 %), under 50 years of age (65 %), and family practice physicians (56 %) (Table 1). Almost half (44 %) had worked in the Fairview system for less than 5 years. PCPs’ support for patient activation varied substantially in the sample. One-third of PCPs had CS-PAM scores below 58.6, a third had scores between 58.6 and 69.9, and the final third had scores between 70.0 and 100.0. Providers in the lowest tercile were least like to indicate positive beliefs about the importance of patient knowledge and involvement in his/her care, compared to those in the middle and highest tercile. Female PCPs had CS-PAM scores 5 points higher (*p* < .05) on average than male PCPs (Table 1). No other provider characteristics were associated with CS-PAM scores.

**Table 1:** Primary care provider (PCP) characteristics and CS-PAM scores

PCP characteristics	Sample demographics	Average
	*n* = 171 (%)	CS-PAM score
Average Score for all PCPs		66.1
Age (in years)		
39 or younger	31.8	65.3
40–49	33.5	67.1
50–59	23.5	65.4
60 or older	11.2	67.2
Years working at fairview		
Less than 5	43.9	65.1
5–10	24.0	67.8
11–20	22.8	66.4
21 or more	9.3	66.4
Type of PCP		
Family practitioner	56.1	66.0
Internist^a^	26.9	65.4
Physician assistant	8.2	71.8
Nurse practitioner	8.8	62.4
Gender		
Male	39.8	62.8
Female*	60.2	68.2

*p < .05

^a^Including double boarded with pediatrics

Bivariate analyses revealed large, positive relationships between CS-PAM and five of the seven self-management support items (Table 2). Compared to PCPs with the lowest CS-PAM tercile scores, those in the highest terciles had two or more times the frequency of “almost always” involving patients in setting the agenda for the visit, checking patient progress towards behavioral goals, asking about patient preferences regarding treatment options, involving the patient in planning how to manage his/her health, and talking to the patient about what he/she can expect from the provider. For example, 47 % of providers in the highest CS-PAM tercile reported making sure the patient was “almost always” involved in setting the agenda for the visit, compared to 36 % for the middle tercile, and 17 % in the lowest tercile.

**Table 2:** Percent of primary care providers who report “Almost always” engaging in chronic illness management support behaviors, based upon CS-PAM scores

	CS-PAM Tercile		
	Lowest	Middle	Highest
	(*n* = 62)	(*n* = 56)	(*n* = 53)
Make sure the patient is involved in setting the agenda for the visit	17.2	36.2	46.5***
Check on progress patient is making toward behavioral goals	18.6	35.6	45.8***
Ask the patient about their personal preferences about treatment options	21.8	33.3	44.8***
Actively involve the patient in problem-solving and planning for how they will manage their health in daily life	13.6	30.5	55.9***
Ask how their chronic illness affects their life	18.2	27.3	54.6
Talked to new patients about what you expect from them as patients	27.3	31.8	40.9
Talked to new patients about what they can expect from you as their clinician	20.5	28.2	51.3*

Tercile 1: 0–58.5, Tercile 2: 58.6–69.9, Tercile 3: 70.0–100.0

*p < 0.05, **p < 0.01, ***p < 0.001

CS-PAM scores were also highly related to the frequency of that PCPs reported using six of the eight strategies to support patient behavior change (Table 3). PCPs in the highest tercile of CS-PAM were six times as likely as those in the lowest terciles to report “very often” bringing the patient back for multiple visits and working with the patient on goal-setting and overcoming barriers. The CS-PAM was also strongly related to PCPs “very often” supporting the patient to work on whatever health goal he/she wants to focus on, having difficult conversations with the patient, providing the patient with detailed after-visit summaries, and attempting not to overwhelm the patient with too many recommendations. The analysis of CS-PAM’s relationship to change in PCPs’ patient panel PAM scores found a moderately positive correlation (*r* = 0.28) (data not shown). Given this finding, no further analyses were conducted to explore the relationship between CS-PAM and changes in patient panel PAM scores.

**Table 3:** Percent reporting “Very often” to using specific strategies to support patient behavior change, based upon CS-PAM score

	CS-PAM Tercile		
	Lowest	Middle	Highest
	(*n* = 62)	(*n* = 56)	(*n* = 53)
Bringing the patient back in for multiple visits to check on progress	10.0	30.0	60.0*
Work with the patient to jointly set behavioral goals and problem-solving to overcome barriers	8.7	34.8	56.5**
Refer the patient to diabetes educators, health coaches, blood pressure nurses or other Fairview support services	23.5	29.4	47.1
Emphasize the serious health risks the patient faces in the future if he or she doesn’t change behavior	25.0	31.3	43.8
Support the patient to work on whatever health behavior goal he or she wants to focus on, regardless of if it will affect quality metrics or not	24.3	27.0	48.7**
Have frank and sometimes difficult conversations with a patient about his or her behaviors	10.7	28.6	60.7^***^
Provide detailed after visit summaries to help a patient remember his or her care plan	24.4	40.2	35.4**
Try not to overwhelm a patient with too many recommended changes	20.0	12.0	68.0***

Tercile 1: 0–58.4, Tercile 2: 58.5–69.9, Tercile 3: 70.0–100.0

*p < 0.05, **p < 0.01, ***p < 0.001

Of the five self-management support items that were related to the CS-PAM in bivariate analyses, three were related in the multivariate models (involving patient in agenda setting, asking patients about personal preferences, involving patients in problem solving) (Table 4). The observed relationships were still large in magnitude. For example, compared to providers in the highest CS-PAM tercile, providers in the lowest tercile had one fifth the odds of involving patients in agenda setting. Those items for which the CS-PAM was not significant in the multivariate models still exhibited trends in which higher CS-PAM PCPs were more likely to “almost always” engage in the behavior than lower CS-PAM respondents.

**Table 4:** Adjusted odds ratios of providers “Almost always” engaging in chronic illness management support behaviors

	Patient involved in agenda setting	Check on progress toward goals	Ask patient about personal preferences	Actively involve the patient in problem-solving	Ask how their chronic illness affects their life	Talk to new patients about expectations for patients	Talked to new patients expectations of PCP
CS-PAM tercile							
Lowest	0.20**	0.27	0.22**	0.10**	0.34	0.56	0.26
Middle	0.62	0.72	0.52	0.30**	0.59	0.78	0.41
Highest	(1.00)	(1.00)	(1.00)	(1.00)	(1.00)	(1.00)	(1.00)
Gender							
Male	1.11	0.88	0.77	0.44	0.39	1.75	1.96
Female	(1.00)	(1.00)	(1.00)	(1.00)	(1.00)	(1.00)	(1.00)
Age (in years)							
39 or younger	1.80	0.65	1.66	0.73	0.20	0.47	0.76
40–49	2.74	0.88	1.58	0.72	0.20	0.80	1.72
50–59	2.30	1.10	1.50	0.78	0.44	0.36	1.23
60 plus	(1.00)	(1.00)	(1.00)	(1.00)	(1.00)	(1.00)	(1.00)
Years working at fairview							
Less than 5	2.12	1.29	1.56	2.56	1.89	1.28	1.55
5–10	1.67	1.53	1.89	2.72	6.37	1.56	1.34
11–20	1.64	1.17	1.45	3.77	5.04	0.64	1.05
21 plus	(1.00)	(1.00)	(1.00)	(1.00)	(1.00)	(1.00)	(1.00)
Type of PCP							
Family practitioners	1.13	2.05	2.33*	5.26**	0.88	1.64	1.11
NP and PAs	0.50	1.80	1.78	3.91*	0.70	2.56	1.90
Internists	(1.00)	(1.00)	(1.00)	(1.00)	(1.00)	(1.00)	(1.00)

*p < 0.05, **p < 0.001

Table 5 shows that the CS-PAM was related to four of the strategies to support patient behavior change (six were related in bivariate analyses). Again, the magnitude of the relationship was large. For example, PCPs in the lowest terciles had less than half the odds of working with the patients to set behavioral goals compared to those in the highest terciles.

**Table 5:** Adjusted odds ratios of providers “Very often” using specific strategies to support patient behavior change

	Bringing patient in for multiple visits	Work with the patient to set behavioral goals	Refer the patient to educators and coaches	Emphasize the serious health risks the patient faces	Support patients behavior goal	Have difficult conversations	Provide detailed after-visit summaries	Try not to overwhelm the patient
CS-PAM tercile								
Lowest	0.10	0.11**	0.33	0.35	0.40	0.11**	0.42	0.17**
Middle	0.39	0.58	0.49	0.54	0.48	0.34*	1.25	0.09**
Highest	(1.00)	(1.00)	(1.00)	(1.00)	(1.00)	(1.00)	(1.00)	(1.00)
Gender								
Male	1.13	0.44	0.75	1.40	0.51	0.75	0.85	0.55
Female	(1.00)	(1.00)	(1.00)	(1.00)	(1.00)	(1.00)	(1.00)	(1.00)
Age (in years)								
39 or younger	1.45	1.94	0.99	0.77	0.49	1.11	0.72	2.50
40–49	0.61	1.06	0.66	0.76	0.51	3.93	2.15	5.09
50–59	0.38	0.97	0.36	0.90	1.60	3.46	0.99	8.28*
60 plus	(1.00)	(1.00)	(1.00)	(1.00)	(1.00)	(1.00)	(1.00)	(1.00)
Years at fairview								
Less than 5	1.29	1.04	1.38	1.20	1.19	1.06	1.32	1.62
5–10	2.05	0.31	0.99	1.09	1.46	0.68	0.91	0.34
11–20	3.94	2.46	1.84	0.97	1.44	0.60	0.67	1.21
21 plus	(1.00)	(1.00)	(1.00)	(1.00)	(1.00)	(1.00)	(1.00)	(1.00)
Type of PCP								
Family practitioners	1.27	1.21	2.19	3.58	1.30	1.93	1.16	2.10
NP and PAs	1.50	1.01	4.00	2.26	0.46	1.22	1.81	0.34
Internists	(1.00)	(1.00)	(1.00)	(1.00)	(1.00)	(1.00)	(1.00)	(1.00)

*p < 0.05, **p < 0.001

The provider characteristic most related to the provider outcome behaviors was the type of PCP. Compared to internists, family practice physicians had twice the odds of asking patients about their personal preferences regarding treatment options. Family practice physicians and non-physicians (nurse practitioners and physician assistants) had five and almost four times the odds, respectively, of actively involving the patient in problem-solving compared with internists.

## Discussion

There is a growing body of evidence that more activated patients have better health outcomes and lower costs than those less engaged in their care [16, 17]. This study explored whether specific clinician beliefs are associated with clinician behaviors and ultimately of greater activation in their patients. The findings indicate that those PCPs whose beliefs are more supportive of patient self-management are more likely to engage with patients with more collaborative and partnership-building behaviors and that the patients of these more supportive clinicians are also more likely to exhibit gains in activation over time than are patients of less supportive clinicians.

Despite a growing literature linking patient activation to a host of quality measures and lower costs, we found substantial variation in PCPs’ beliefs about patient activation. Even in an accountable care organization, where there is a strong emphasis on engaging patients, many PCPs had less favorable beliefs about the patients’ role. Surprisingly, PCPs’ beliefs, as measured by the CS-PAM were not associated with their patient panel characteristics. Nor were they related to the PCPs’ own demographic characteristics, which was similar to the findings of Hibbard and colleagues [6]. The one exception was that female PCPs had more positive beliefs (as measured by the CS-PAM) than male PCPs.

We found the measure of PCPs’ beliefs to be strongly related to PCPs’ report of the frequency of behaviors supportive of patient self-management and behavior change. The strategies most commonly employed by PCPs in each tercile of CS-PAM were quite revealing: providers with CS-PAM scores in the highest tercile were most frequently trying not to overwhelm patients with too much behavior change; providers with middle tercile CS-PAM scores were most frequently providing detailed after visit summaries–a relatively passive behavior; providers with the lowest tercile scores were most frequently emphasizing the serious health risks if the patient did not change behavior. All these provider behaviors have been previously described in the literature [11]. In this study we were able to connect these behaviors to provider beliefs. Indeed, the CS-PAM score seems to differentiate clinicians on the efficacy of the strategies they most often use to motivate behavioral changes.

Healthcare organizations can assess their own clinicians, measure their CS-PAM scores, and design interventions to help low scoring clinicians gain knowledge and skills in supporting the patients’ role. Educators training the next generation of primary care providers should emphasize not just the importance of supporting patient self-management but also the acquisition of skills and the use of strategies that are most effective in encouraging patient behavior changes. Prior efforts to educate providers on supporting patient self-management during medical training and afterwards have been shown to be effective [18].

The generalizability of the study’s findings are limited by the fact that they are drawn from a single healthcare organization. In addition, the findings are based on a cross-sectional analysis, limiting our understanding of the temporal ordering of beliefs and behaviors. We assume the beliefs precede the behaviors, but we cannot confirm that with cross-sectional survey data. Further, the assessment of clinician behaviors we employed in the study is based on self-reports, and may not reflect actual behavior. However, the patient panel changes in level of patient activation is derived from a different source and the relationship with CS-PAM was consistent with that found with the clinician behavior items. Finally, the patient panel changes in level of patient activation were only available for a portion of all patients and a portion of the clinicians. Future research should examine the relationship between PCPs’ beliefs in the importance of patient involvement in self-management and changes in patient activation using a larger sample of providers with more patients in their panel.

## Conclusions

The findings of this study highlight the important role PCPs can play in increasing patient engagement in their care. The findings also reveal the high degree of variation among PCPs in terms of their beliefs and their behaviors in support of patient self-management—all within one accountable care organization. This type of variation among primary care providers appears to be associated with variations in patient activation, within provider patient panels, and likely patient outcomes. Reducing this variation among PCPs is important for accountable care organizations because they rely on patient loyalty to keep patients within their system, and are seeking to reduce costs and improve outcomes, in part, by engaging and activating patients. Having primary care providers who are uniformly and consistently supportive of patient self-management will be a strategic competitive advantage in the post-affordable care act world.

## Appendix

**Table 6:** Items for Clinician Support for Patient Activation Measure

As a clinician, how important is it to you that your patients with CHRONIC CONDITIONS:	
1.	Are able to take actions that will help prevent or minimize symptoms associated with their health condition
2.	Are able to maintain lifestyle changes needed to manage their long-term condition
3.	Understand which of their behaviors make their condition better and which ones make it worse
4.	Can follow through on medical treatments they need to do at home
5.	Know what each prescribed medication does
6.	Bring a list of questions when they come to the clinic
7.	Are able to determine when they need to go to a medical professional for care versus when they can manage the problem on their own
8.	Are able to work out solutions when new situations or problems arise with their health condition
9.	Want to be involved as a full partner with you in making decisions about care
10.	Tell you concerns they have about their health even when you do not ask
11.	Want to know what procedures or treatments they will receive and why before the treatments are performed
12.	Understand the different medical treatment options available for their long term condition
13.	Look for trustworthy sources of information about their health and health choices such as on the web, news, or books
